# The clinically excellent primary care physician: examples from the published literature

**DOI:** 10.1186/s12875-016-0569-x

**Published:** 2016-12-13

**Authors:** Kimberley Lee, Scott M. Wright, Leah Wolfe

**Affiliations:** Division of General Internal Medicine, Johns Hopkins Bayview Medical Center, Johns Hopkins University School of Medicine, 5200 Eastern Ave., Mason F. Lord Center Tower, 2nd Floor, Baltimore, 21224 MD USA

**Keywords:** Clinical excellence, Primary care

## Abstract

Clinical excellence is the ultimate goal in patient care. Exactly what the clinically excellent primary care physician (PCP) looks like and her characteristics have not been explicitly described. This manuscript serves to illustrate clinical excellence in primary care, using primarily case reports exemplifying physicians delivering holistic and patient-centred care to their patients. With an ever increasing demand for accessible and accountable health care, an understanding of the qualities desirable in primary care providers is now especially relevant.

A literature review was conducted to identify compelling stories showing how excellent PCPs care for their patients. In the 2397 published works reviewed, we were able to find case reports and studies that exemplified every domain of the description of clinical excellence proposed and published by the Miller Coulson Academy of Clinical Excellence (MCACE). After reviewing these reports, the authors felt that the domains of excellence, as described by the MCACE, are practically applicable and relevant for primary care physicians. It is our hope that this paper prompts readers to reflect on clinical excellence in primary care.

## Background

Excellence in western medicine was historically founded on knowledge with the application of anatomy and physiology of the care of patients. The approach to care had been disease-oriented and problem-based [1, 2]. Contemporary medicine has added to these earlier priorities a genuine commitment to patient-centered and biopsychosocial approaches to the care of patients [3]. In characterizing clinical performance among primary care physicians (PCP), critical work by The Leeuwenhorst Group, The Royal College of General Practitioners, and The World Organization of Family Doctors (WONCA) have been effective on many fronts, including convening experts to consider and define best practices for medical professionals to provide comprehensive care for both individuals and the communities they are serving [4–7].

More recent scholarly work, not concentrating solely on generalism and primary care, resulted in a comprehensive description of the elements that contribute to clinical excellence [8]. This schema for clinical excellence has been applied to different medical subspecialties (such as cardiology [9] and psychiatry [10]) to delineate what clinical excellence looks like for physicians across varied fields of practice [11–14]. In this manuscript, we set out to identify examples of clinically excellent primary care physicians performing along ach of the facets of clinical excellence in the framework.

The Miller-Coulson Academy of Clinical Excellence (MCACE) at Johns Hopkins University School of Medicine was established to recognize excellence in patient care for the benefit of the patients and communities served by the institution [15]. The MCACE works to highlight and perpetuate the ideals of clinical excellence through many distinct programs geared towards patients, medical students, residents, and practicing clinicians. Rigorous methods, which include evaluation by external referees and an internal selection committee—with both parties using validated metrics to assess the 50+ page clinical portfolios compiled by the clinicians, determine which nominees are inducted as new members (this consideration occurs once each year and membership has always been extended to fewer than 50%).

Patients seen in primary care practices genuinely appreciate PCPs who are patient-centred, and those who “listen, explain, and are thorough” [16, 17]. In contrast, when patients feel that their insights are being ignored by their PCPs, they have become passive and more distraught about their illness [8]. Dissatisfaction with providers has been shown to be associated with increased healthcare utilization [3]. Because PCPs are ‘generalists’, [18] they must be continuously ready for anything and everything that their patients bring to them. Clearly, PCPs have an integral role in the health of individual patients and the communities that they serve—as patients’ first and main contact with the system [19].

This manuscript highlights papers from the literature, mostly published case reports, which exemplify the core domains of clinical excellence as they are played out by practicing PCPs caring for their patients. It is hoped that this manuscript promotes reflection on clinical excellence in primary care.

## Definition of primary care physicians and characterization of clinical excellence

Primary care physicians are central and core components of any high quality health care delivery system [20, 21]. These physicians provide care longitudinally and continuously [22]. Further, they are holistic in their approach and attend to the whole person—rather than focusing in a more disease-oriented manner [23]. Since we have already published a paper about clinical excellence in pediatrics, [14] this paper will address clinical excellence in PCPs caring for adults.

Multiple methodological approaches, including a systematic review of the literature as well as both quantitative and qualitative studies, were used to comprehensively characterize clinical excellence before establishing the Miller-Coulson Academy of Clinical Excellence (MCACE) at Johns Hopkins in Baltimore [8, 15]. The following description of *clinical excellence* is used for all MCACE related initiatives:

- Achieving distinction in 6 areas as they relate to patient care:
  - communication & interpersonal skills
  - professionalism and humanism
  - diagnostic acumen
  - skillful negotiation of the healthcare system
  - knowledge
  - scholarly approach to clinical practice, and
  - Exhibiting a passion for patient care

## Methods for identification and selection of examples to be highlighted

A senior medical informationist from our library was enlisted to help with finding examples of PCPs and primary care encounters to be highlighted in this paper. The authors and the informationist performed a search of PubMed for the years of 1962 through November 2013. Initial broad searches combined the keywords of ‘primary care’ or ‘primary care physician’ and ‘clinical excellence’; limiting the output primarily to case reports. Searches were also narrowed by using the limits of ‘humans’ and ‘English’. In subsequent searches, the term ‘clinical excellence’ was replaced by each of the components of the definition bulleted above (e.g. diagnostic acumen). Using these parameters and limits, a total of 2397 unique published case reports and studies were identified. From these articles, we selected those for which the abstract or summary indicated that the paper pertained to the provision of excellent primary care. In the end, at least 10 papers for each component of the definition were identified. These articles were then read in their entirety to fully understand the patient’s story and the medical care that was delivered. A majority of these papers were dismissed because there was no description of the interaction between the physician and patient, most were alternatively focused on the biomedical aspects of the care provided. The authors, one of whom directs the MCACE and a second who was one of the inaugural members, discussed the remaining candidate case reports and considered the extent to which each case represented superlative quality performance in one of the domains. The cases to serve as exemplars were selected by consensus, with attention to ensure that all were truly from primary care settings.

## Application of the description of clinical excellence to primary care physicians

To illustrate how distinction in each of these domains is exemplified by primary care physicians, the authors selected 8 case reports and 9 other exemplary illustrations from the literature. Each case or study demonstrates how superior performance in a particular domain of clinical excellence enables clinicians to serve and provide the best possible care for patients.

### Communication and interpersonal skills

The PCP’s communication and interpersonal skills are critical for promoting successful physician-patient relationships [24]. Giving patients the opportunity to share their stories and perspectives, through active listening, improves trust, adherence, and some biophysical outcomes. Empirical research has shown lower blood pressures, better control of hemoglobin A1c, and faster symptom resolution when patients are encouraged to share their illness narratives [25]. Patient-centred communication has been shown to transcend race and gender concordance, thereby allowing for additional dimensions of commonality [26, 27].

An 80-year old man did not agree with his primary care physician’s recommendations regarding the testing needed for his rectal bleeding [28]. Discussions with the patient enabled the primary care doctor to learn about the patient’s underlying concerns and sources of confusion regarding the recommendations. The doctor discovered that once the patient’s bowel movements no longer contained gross blood, the patient no longer saw the point of testing. The case report describes how the clinician explained his rationale, checked for the patient’s understanding, and then came to a mutually agreeable plan of action that was in line with and respectful of the patient’s wishes. The patient was interviewed after this encounter and he reported that only when the doctor’s suggestions made sense to him, was he willing to follow through. Partnering with patients to gain an understanding of their priorities in establishing goals of care, a key elements of patient-centred care, is wise and appreciated by patients [29].

A second case illustrates that productive dialogue with patients can influence patient behaviors and outcomes [30]. Joanne was a 35-year-old woman with chronic back pain. The case report depicts one visit with her PCP wherein she told him that she could not go on vacation because of the pain. Her PCP in turn encouraged her to go and suggested that together they modify her pain control regimen to alleviate additional pain brought on by travelling. Joanne explained that she was agreeable because her PCP took the time to engage in thoughtful guidance and counseling. She described appreciating her PCP’s non-judgmental and understanding approach, and “that he talked it through with me.” Chronic pain is a highly prevalent condition [31]. Treatment of pain can be a source of frustration for both patients and PCPs. Open and honest communication about goals of care can strengthen the therapeutic alliance.

### Professionalism and humanism

Professionalism and humanism support the development and maintenance of strong longitudinal physician-patient relationships in primary care. The humanistic physician is empathetic, dedicated to service, and focused on the patient who has the disease rather than the disease that the patient has. Physician empathy has been empirically associated with improved clinical outcomes [32]. The clinically excellent primary care physician behaves professionally and humanistically consistently, both when faced with demanding situations and when encountering routine matters.

Ms. X, a 28-year-old woman, presented to an emergency room with upper respiratory tract infection symptoms [29]. She was diagnosed with allergic rhinitis and treated. Over the next 3 years, Ms. X frequented emergency departments and urgent care clinics with similar complaints. During one 6-month period, she saw 17 different health-care providers and her medications costs were over $750 per month. Ms. X developed steroid-induced diabetes. Then, a committed PCP agreed to partner with Ms. X to comprehensively care for her holistically, with great attention to ensuring access and continuity. A contract used to capture elements of the agreement and both parties concurred that she would direct all telephone calls and requests for urgent visits to the PCP. Access to the physician was an integral part of this plan – the patient had to be able to reliably get in touch with a member of the team. All members of the team were understanding and consistently treated the patient compassionately. Even though this approach meant increased calls and visits to the PCP initially, professionalism and humanism (both manifested as a genuine commitment to patient welfare) required the PCP to make himself available to the patient. This intervention resulted in decreased emergency room visits, decreased medication costs, and an overall improvement of her health. This physician’s behavior exemplifies that compassion and responsiveness to the needs of the patient can be life changing.

### Diagnostic acumen

A primary care provider with superior diagnostic acumen is often on the receiving end of unofficial consults regarding patients with perplexing symptoms and uncertain diagnoses. Such physicians are skilled in the science and art of using information gathered from the history and physical exam to arrive at the correct diagnosis [8, 9].

Because a skillful aptitude in diagnostic acumen can be seen in the cases shared in other sections of this paper, 2 studies are presented here. Nendaz and colleagues set out to determine factors that contribute to diagnostic accuracy [33]. Senior medical students, junior residents, and experienced general internists each assessed the same seven patient cases. These standardized patients had chief complaints that are common in primary care, varying from cough to arthritis. Factors associated with making the correct diagnosis included clinical experience, thinking of a larger differential diagnosis initially, and then eliminating highly unlikely considerations as the clinical scenario unfolds. Not unexpectedly, the diagnostic accuracy of the internists was significantly greater than that of residents, and medical students. *Testing clinical hypotheses* was described to be at the heart of diagnostic acumen.

Another study enlisted 21 primary care physicians with an average of 20 years’ experience to assess their clinical reasoning [34]. They were given 10 clinical scenarios that were validated to distinguish across levels of clinical reasoning. The physicians were asked for (i) the most likely diagnoses, and (ii) the specific features which led to them to their conclusion. Those who were most skilled often made the correct diagnosis with less clinical data than others. Expertise in collecting and integrating pertinent information, reflecting upon and reconciling pieces that do not fit, and developing sound unifying diagnoses are fundamental skills necessary to be a master diagnostician.

### Skillful negotiation of the healthcare system

Health care systems have become increasingly fragmented with many providers, sites of care, and record systems. The system may have cracks that can make the navigation arduous for patients during their quest for high quality care. Specific populations, for example those with limited health literacy, are especially vulnerable to receiving suboptimal care. Those aspiring to deliver excellent patient care to all patients must be effective advocates, and knowledgeable about the resources available for patients. On the frontlines, PCPs are instrumental in helping patients to negotiate our complicated healthcare landscape.

A 64-year-old female and her PCP had been working together to alleviate her chronic hip pain, partially attributable to arthritis [35]. The search for an effective analgesic regimen was limited by the patient’s insurance and financial situation. Further, she had intolerable side effects to several attempted therapies. The PCP and his team were ultimately able to enroll the patient in a program that would pay for the non-generic (brand name) medication that the patient wanted, and that would otherwise have been too expensive for her. Social determinants, particularly the socioeconomic status of individuals, can have a great impact on health, wellness, disease, and infirmity.

A practice-based solution that masterfully supports vulnerable patients and their family members is called *Health Leads. Health Leads* mobilizes community resources to help patients with challenges that affect health, such as nutrition, shelter, and vocation [36]. This organization partners with primary care providers to address such social determinants of health, specifically those that are not customarily addressed by PCPs in their offices. This holistic, patient-centred care, which often involves proactive problem solving around the issues that matter most to the patient, epitomizes clinical excellence.

### Knowledge

Knowledge may be considered to be the cornerstone of clinical excellence. The early part of medical school is devoted primarily to the acquisition of medical knowledge, and this quest as a lifelong learner never ends for the clinically excellent physician. The domains of diagnostic acumen and scholarly approach to the practice of medicine rest on the foundation of knowledge; or at the very least, an understanding of what knowledge is required to solve a clinical problem and the ability to find, interpret, and apply this information.

The following case illustrates how a PCP’s knowledge and memory of clinical information read years earlier proved helpful when faced with a puzzling scenario. A 44-year-old male suffered from asthma, which did not respond to bronchodilators [37]. He had been followed by a pulmonologist who attributed his symptoms to his history of smoking socially in college. He initially presented to his PCP with questions about becoming a liver donor for his father. His father had cirrhosis thought to be due to alcohol consumption, though he later tested positive for Alpha-1 Antitrypsin (AAT) deficiency. With this history, the PCP’s suspicion for AAT deficiency led to testing which confirmed that this patient was also AAT deficient. This PCP’s knowledge of the association of AAT deficiency with both liver and pulmonary pathology, and her ability to discern salient aspects of the patient’s clinical presentation, prevented further delay in diagnosing this chronic, progressive disease. Ultimately, appropriate therapy was delivered.

### Scholarly approach to clinical practice

Medical information is constantly evolving and clinically excellent primary care doctors remain abreast of discoveries. Further, after critically appraising newly published studies, they extract the relevant clinical material and apply it effectively in caring for patients. Using actual patient data to improve one’s clinical performance, as exemplified by the case below, also represents a scholarly approach to patient care.

A primary care doctor resolved to improve the diabetes care he provided after discovering that his performance in this area, as measured by clinical outcomes, was substandard [38]. When strategies implemented indiscriminately failed to yield significant improvements, he decided to pursue a more scholarly approach to achieve the goal. Using a variation on a published model for quality improvement, [39] he made changes to his practice. These efforts were successful in dramatically increasing the proportion of his patients who met goals related to diabetes care and cardiovascular risk factors. Additionally, the measures were adopted his colleagues, thereby expanding the influence to benefit even more patients.

Both individual PCPs and generalist societies or organizations continue to be engaged in high quality empiric research to discover optimal approaches for delivering primary care [40, 41]. Further, health systems and postgraduate educational agencies are relying on documented compliance with curricula for continuous professional development (or continuing medical education [CME]) to ensure that physicians remain informed regarding best practices [42–45].

### Exhibiting a passion for patient care

Physicians who are passionate about primary care love the privilege providing longitudinal care to their patients and forging meaningful relationships with them over time. They relish the responsibility of being the tip of the health care system’s arrow, not only granting timely access to expertise but also being the first to consider patients’ concerns while promoting wellness and the prevention of illness. These driven individuals are often innovators.

Dr. Bob Paeglow is a primary care internist who came to medicine later than most, enrolling in medical school at the age of 36 years and hoping to make the world a better place [45]. He practices in a disadvantaged neighborhood in Albany, N.Y., often personally financing his patients’ medical care, and foregoing salary regularly. Commonly known as Dr. Bob in the community, Dr. Paeglow repeatedly goes above and beyond standard medical care to support his patients; he threw a birthday party for the daughter of a patient with advanced colon cancer, bought Christmas presents for the child of one of his patients who was unable to do so, and paid for psychologist visits for a new patient who was suffering with depression.

Similarly, Dr. Regina Benjamin has devoted her life to the care of the patients in a small town in rural Alabama [46]. Shortly after graduating from medical school, she founded a clinic to take care of patients in an underserved fishing village, 80% of whom live below the federal poverty level. She physically helped in the rebuilding of this clinic when it was destroyed by a hurricane. During this time of its reconstruction, she cared for her patients visiting them in their homes.

Both role model physicians described above are passionate about primary care and they are incredibly generous—constantly giving to their individual patients, and the community at large.

## Discussion and conclusions

Describing excellence in any area, from athletic to vocational, is a complex endeavor largely because there are numerous factors that contribute to ‘excellence’. The realization of excellence requires investing much time, engaging in deliberate practice, and maintaining precise focus on the goal.

This manuscript describes many domains of clinical excellence, as they are applicable to primary care physicians. Primary care physicians collaborate within multidisciplinary teams; [47, 48] such partnerships make it possible for them to thoroughly support individual patients and communities. Still, it is imperative to carefully consider what excellence means and looks like for the team leader - the primary care physician. The influential and thoughtful work by The Leeuwenhorst Group and The Royal College of General Practitioners focus largely on thresholds for competence and what are expected minimum norms [49–52]. Competence represents the floor above which all PCPs must surpass. Excellence among primary care physicians, by contrast, is the aspirational ceiling that many, but not all, strive to reach. In collating case examples from the published literature of PCP delivering masterful care, we hoped to highlight the adjacent possible [53].

Like the other papers in this series, most focusing on specialists rather than generalists, the authors were able to find descriptions of exemplary PCPs modeling with distinction in each of the domains of clinical excellence in the published literature. The utility of this manuscript and its clinical implications will be intricately tied to how it is used by readers. It is our hope that this paper will promote reflection about the domains and specific examples above; in so doing, readers will think about their own approaches and proficiencies. This reflection on practice can result in greater self-awareness and a deeper understanding of strengths, weaknesses, and professional goals in primary care [54].
